# Supplementary material for: Spontaneous crystallization of strongly confined CsSnxPb1-xI3 perovskite colloidal quantum dots at room temperature
Source: Nat Commun. 2024 Feb 21;15:1609. doi: 10.1038/s41467-024-45945-1 (PMC10881968; doi:10.1038/s41467-024-45945-1)
Supplement: Supplementary file 1 — Supplementary Information [file 41467_2024_45945_MOESM1_ESM.pdf]

Supplementary Information

Spontaneous crystallization of strongly confined $CsSn_xPb_{1-x}I_3$ perovskite colloidal quantum dots at room temperature

Louwen Zhang[1,2,3], Hai Zhou[1]*, Yibo Chen[4], Zhimiao Zheng[2], Lishuai Huang[2], Chen Wang[2], Kailian Dong[2], Zhongqiang Hu[3], Weijun Ke[2] & Guojia Fang[2]*

[1]*International School of Microelectronics, Dongguan University of Technology, Dongguan 523808, Guangdong, P. R. China*

[2]*Key Lab of Artifcial Micro- and Nano-Structures of Ministry of Education of China, School of Physics and Technology, Wuhan University, Wuhan 430072, P. R. China*

[3]*School of Electronic Science and Engineering, Xi'an Jiaotong University, Xi'an 710049, P. R. China*

[4]*Institute of Fluid Physics, China Academy of Engineering Physics, Mianyang 621900, P. R. China*

* Corresponding Author, E-mail addresses: hizhou@dgut.edu.cn (H. Zhou); gjfang@whu.edu.cn (G. Fang)

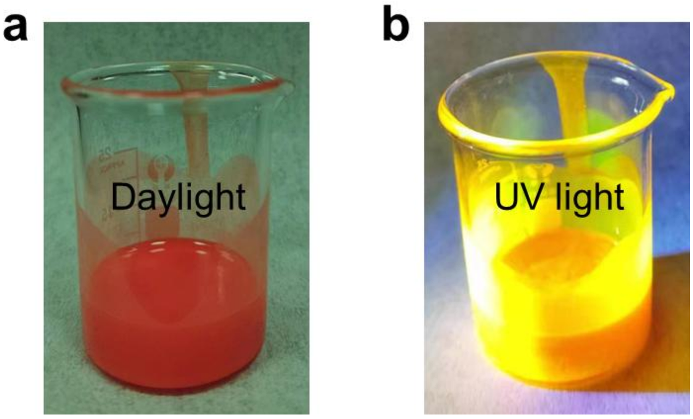

Supplementary Fig. 1 | Photographs of the as-synthesized pure-iodine all-inorganic $CsSn_xPb_{1-x}I_3$ mixed-cation perovskite colloidal quantum dots (QDs). The crude QD colloidal solution under **(a)** daylight and **(b)** UV light.

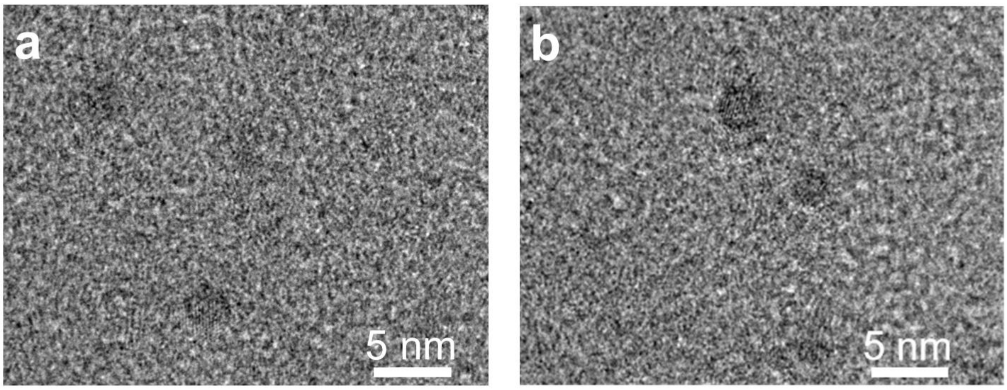

Supplementary Fig. 2 | (a, b) HRTEM images of pure-iodine all-inorganic $CsSn_{0.09}Pb_{0.91}I_3$ perovskite QDs.

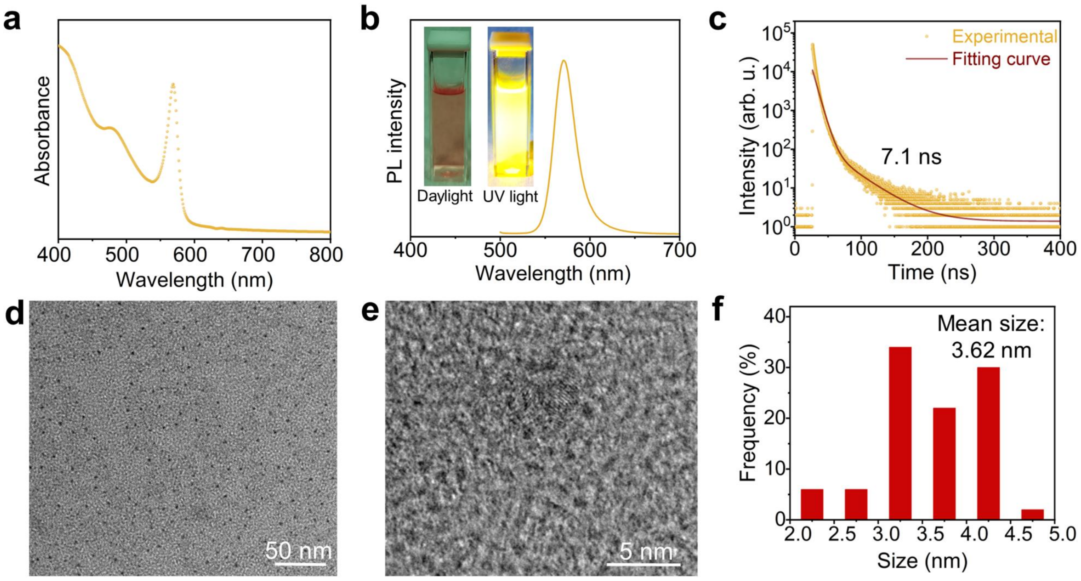

Supplementary Fig. 3 | Optical properties of FA-based Sn-Pb iodide organic-inorganic hybrid perovskite QDs. (a) Steady-state absorption, **(b)** PL spectra and **(c)** PL decay curve of hybrid perovskite QDs. Insets of **(b)**: photographs of the diluted QD colloidal solution under daylight and UV light, respectively. **(d)** TEM image and the corresponding **(e)** HRTEM image and **(f)** size distribution (QD statistical quantity: 50) of hybrid perovskite QDs. The statistical result show that the as-prepared Sn-Pb hybrid perovskite QDs exhibit sub-5 nm size with strong size confinement. The actual proportion of Sn/(Sn+Pb) in the product is 10.8%, which is determined by ICP-OES. Source data are provided as a Source Data file.

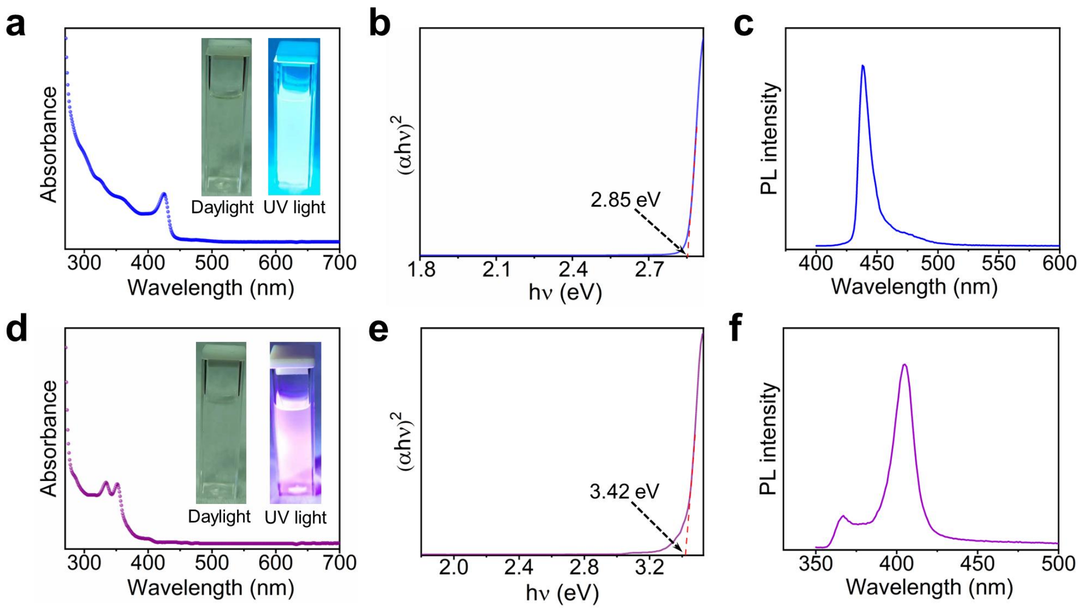

Supplementary Fig. 4 | Optical properties of Br- and Cl-based all-inorganic Sn-Pb perovskite QDs. (a) Steady-state absorption spectrum, **(b)** Tauc plot, and **(c)** PL spectrum of Br-based all-inorganic Sn-Pb perovskite QDs. **(d)** Steady-state absorption spectrum, **(e)** Tauc plot and **(f)** PL spectrum of Cl-based all-inorganic Sn-Pb perovskite QDs. Insets of **(a, d)**: the corresponding photographs of the diluted QD colloidal solution under daylight and UV light, respectively. The small bumps for both Br (at 500 nm) and Cl (at 410 nm) samples in **(a, d)** are attributed to the diversity of NC sizes (high size polydispersity), which is consistent with the emission peak positions in the PL spectra. Source data are provided as a Source Data file.

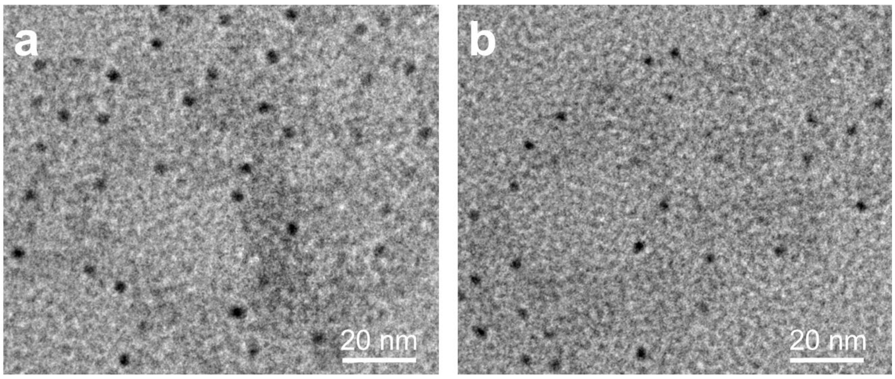

Supplementary Fig. 5 | (a, b) TEM images of $CsPbI_3$ perovskite QDs.

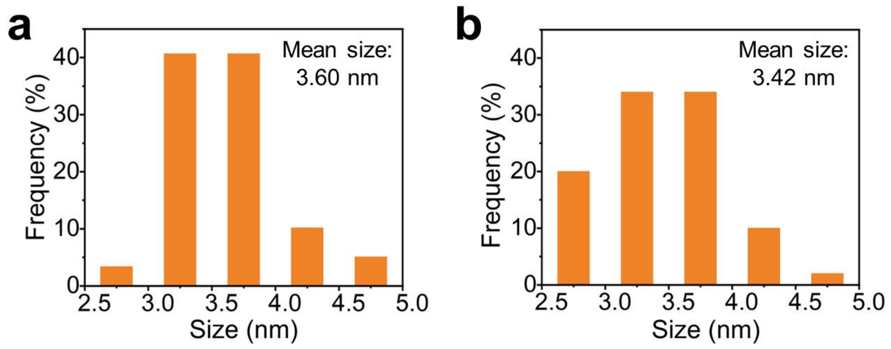

Supplementary Fig. 6 | Size distribution (QD statistical quantity: 50) of perovskite QDs. (a) $CsPbI_3$ QDs. **(b)** $CsSn_{0.09}Pb_{0.91}I_3$ QDs. The statistical results indicate that the as-prepared perovskite QDs exhibit a uniform size distribution and sub-5 nm size. Source data are provided as a Source Data file.

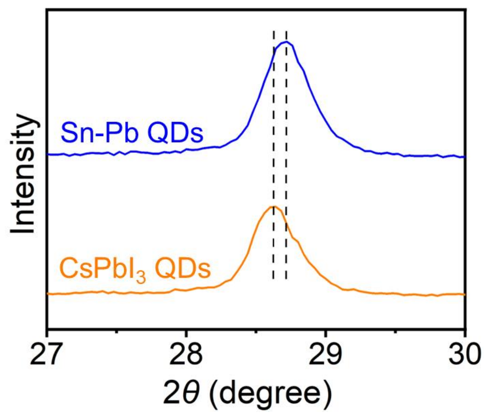

Supplementary Fig. 7 | Partial enlarged view for XRD patterns of $CsPbI_3$ QDs and $CsSn_{0.09}Pb_{0.91}I_3$ QDs. Source data are provided as a Source Data file.

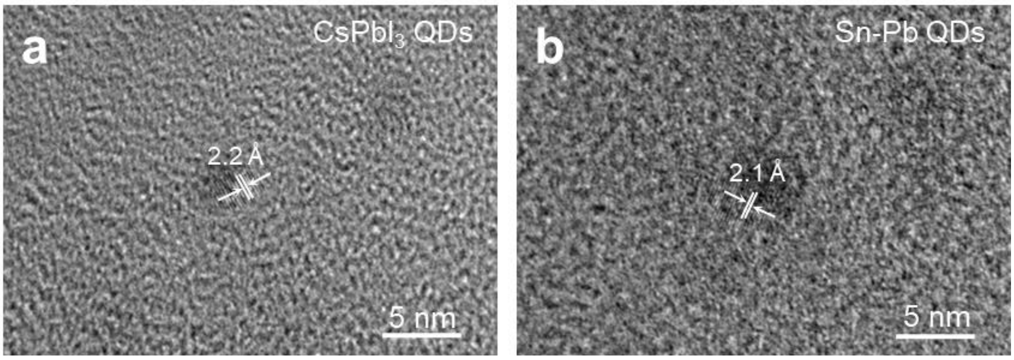

Supplementary Fig. 8 | HRTEM images of perovskite QDs. (a) $CsPbI_3$ QDs. **(b)** $CsSn_{0.09}Pb_{0.91}I_3$ QDs. This shows the lattice contraction of the perovskite structure with the incorporation of stannous ions.

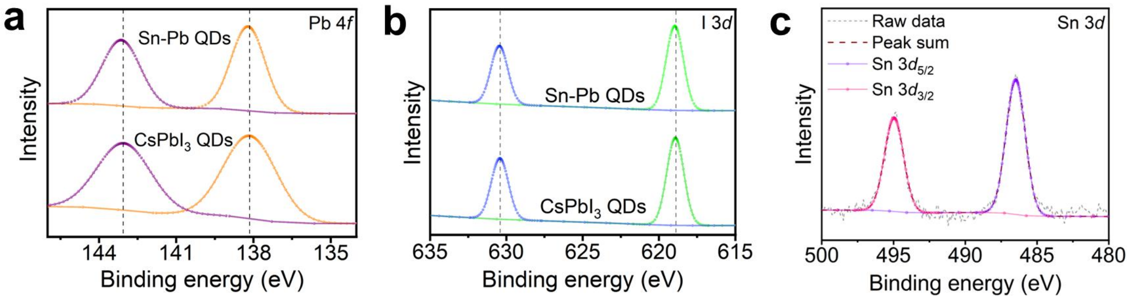

Supplementary Fig. 9 | XPS characterization of $CsPbI_3$ and $CsSn_{0.09}Pb_{0.91}I_3$ QDs. High-resolution XPS spectra corresponding to **(a)** Pb 4*f*, **(b)** I 3*d*, and **(c)** Sn 3*d*. Owing to the lack of solvent protection and large surface area of QDs, the preparation process of Sn-Pb QD film sample for XPS test was carried out in a glovebox to avoid oxidation. Source data are provided as a Source Data file.

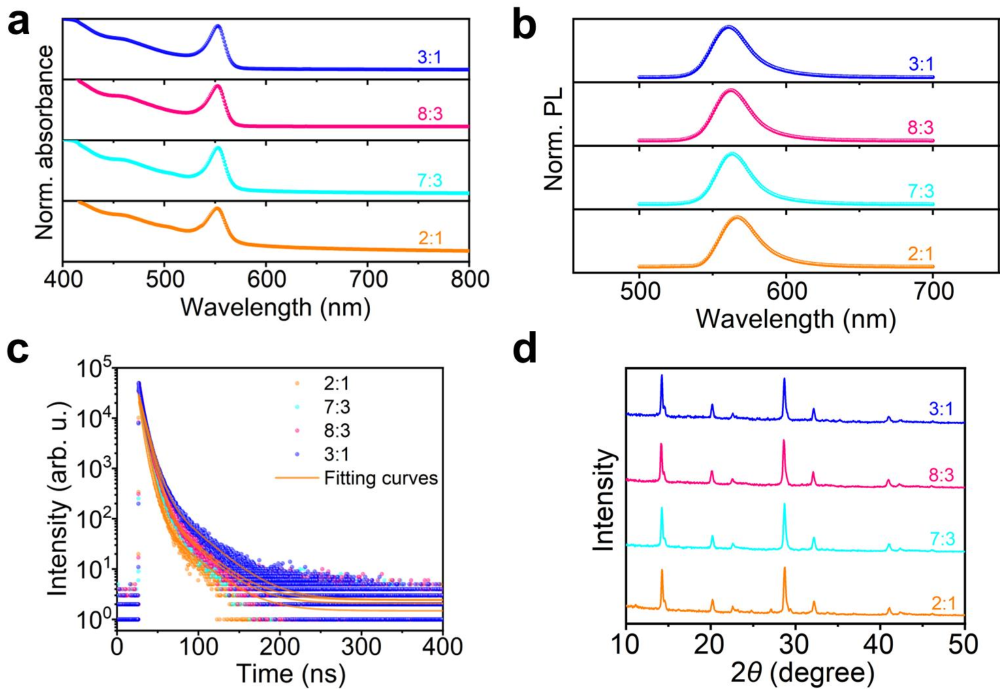

Supplementary Fig. 10 | Optical properties and XRD analysis of pure-iodine all-inorganic Sn-Pb perovskite QDs with different Pb/Sn ratio. (a) Steady-state absorption. **(b)** PL spectra. **(c)** PL decay curves. **(d)** XRD patterns. Source data are provided as a Source Data file.

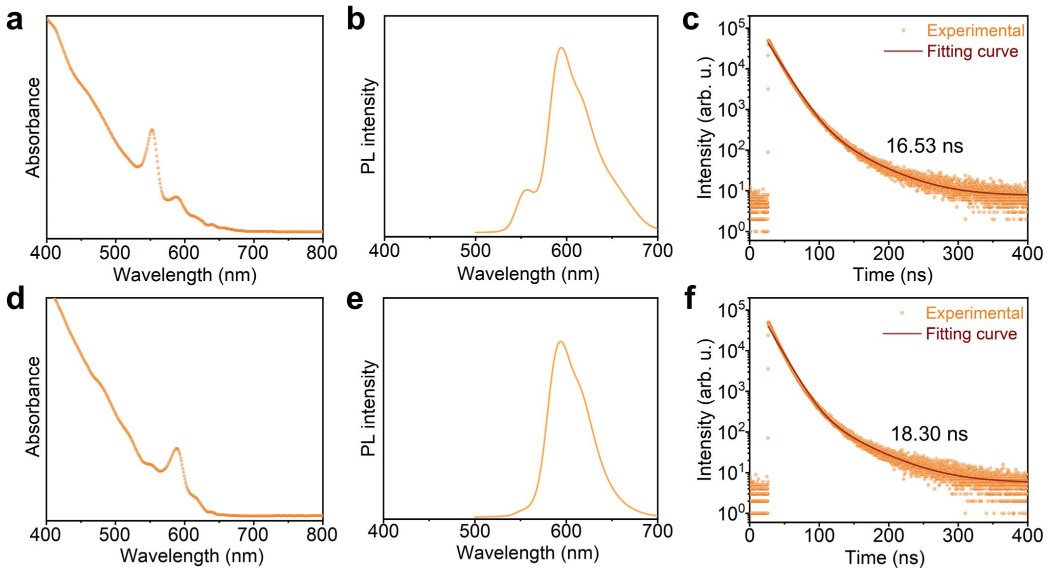

Supplementary Fig. 11 | Optical properties of pure-iodine all-inorganic Sn-Pb perovskite QDs synthesized at 120 ℃ for (a-c) 3 min and (d-f) 6 min. (a, d) Steady-state absorption. **(b, e)** PL spectra. **(c, f)** PL decay curves. Source data are provided as a Source Data file.

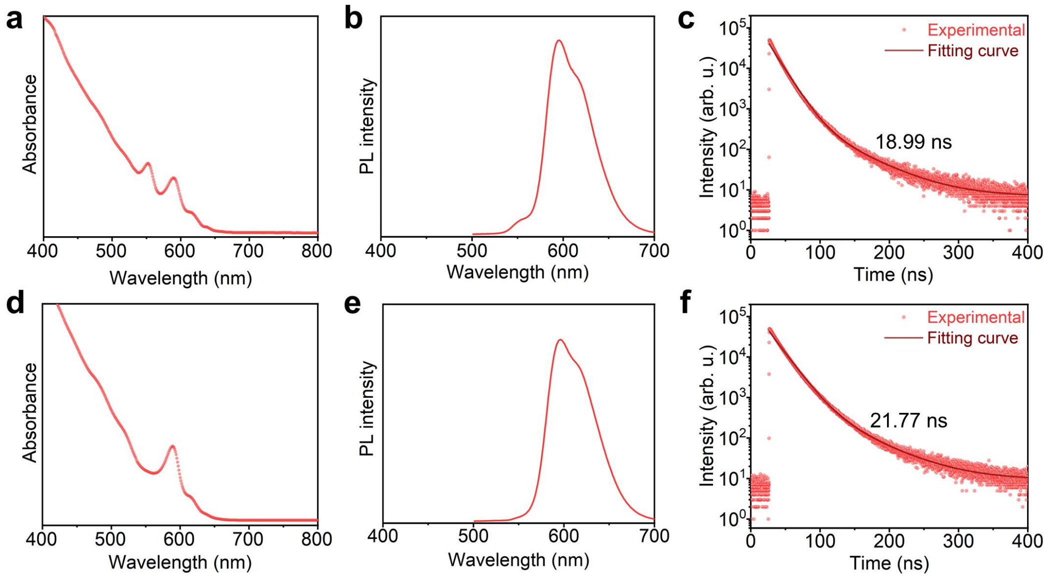

Supplementary Fig. 12 | Optical properties of pure-iodine all-inorganic Sn-Pb perovskite QDs synthesized at 180 ℃ for (a-c) 3 min and (d-f) 6 min. (a, d) Steady-state absorption. **(b, e)** PL spectra. **(c, f)** PL decay curves. Source data are provided as a Source Data file.

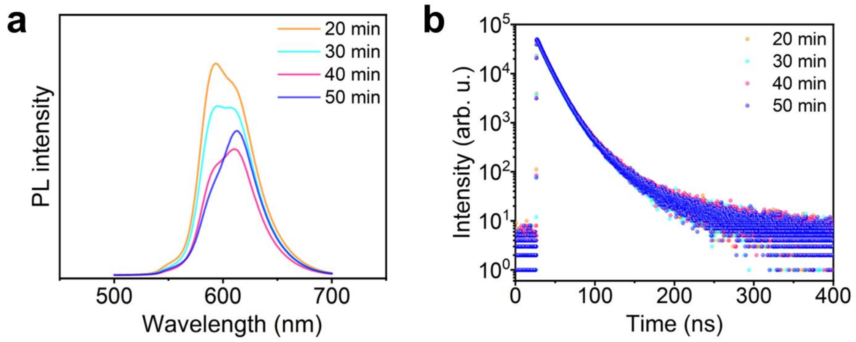

Supplementary Fig. 13 | Optical properties of pure-iodine all-inorganic Sn-Pb perovskite QDs synthesized at 120 ℃ for the extended heating times. (a) PL spectra. **(b)** PL decay curves. Source data are provided as a Source Data file.

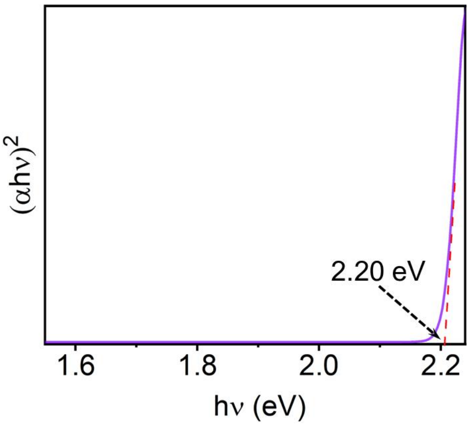

Supplementary Fig. 14 | Tauc plot of pure-iodine all-inorganic $CsSn_{0.09}Pb_{0.91}I_3$ QDs according to the absorption spectrum. Source data are provided as a Source Data file.

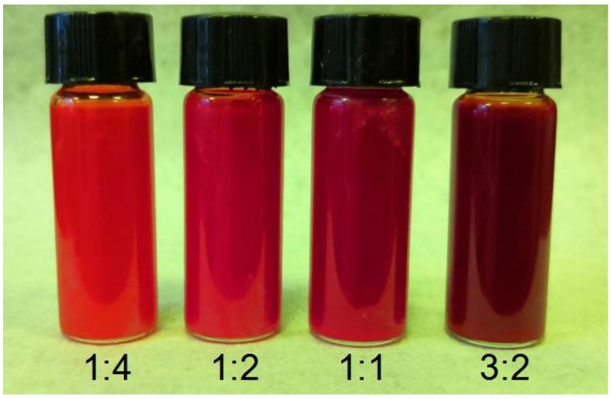

Supplementary Fig. 15 | Photographs of pure-iodine all-inorganic Sn-Pb perovskite colloidal solutions with varying Cs/Pb feed molar ratio under daylight.

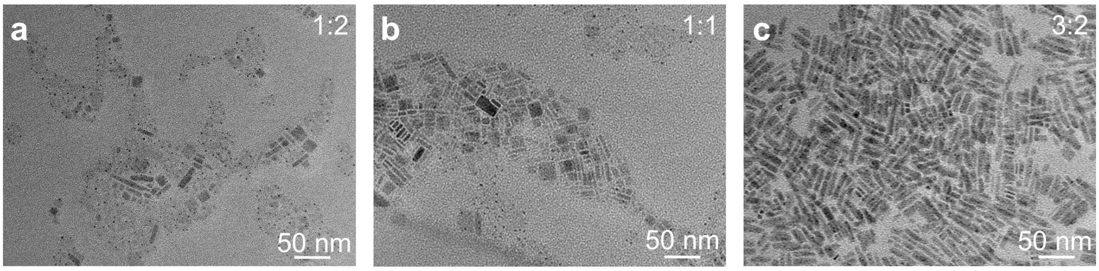

Supplementary Fig. 16 | TEM images of pure-iodine all-inorganic Sn-Pb perovskite QDs with varying Cs/Pb feed molar ratio. (a) Cs/Pb ratio of 1:2. **(b)** Cs/Pb ratio of 1:1. **(c)** Cs/Pb ratio of 3:2.

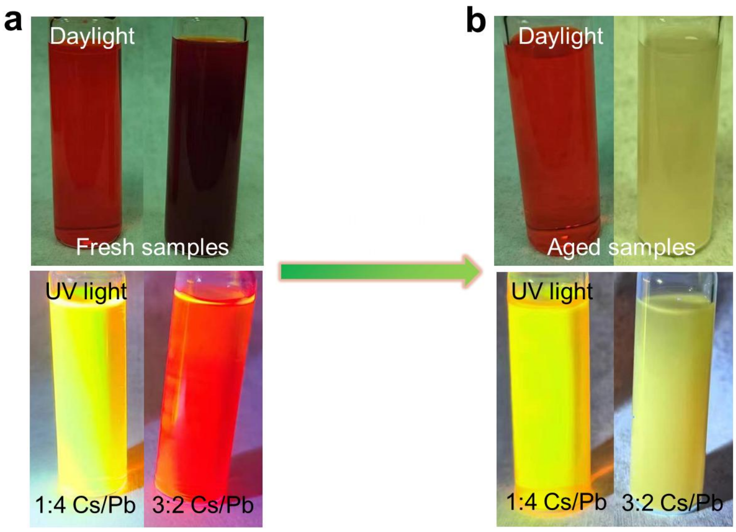

Supplementary Fig. 17 | Photographs under daylight and UV light showing the colloidal stability in air for the samples with Cs/Pb ratios of 1:4 and 3:2. (a) Fresh samples. **(b)** Aged samples.

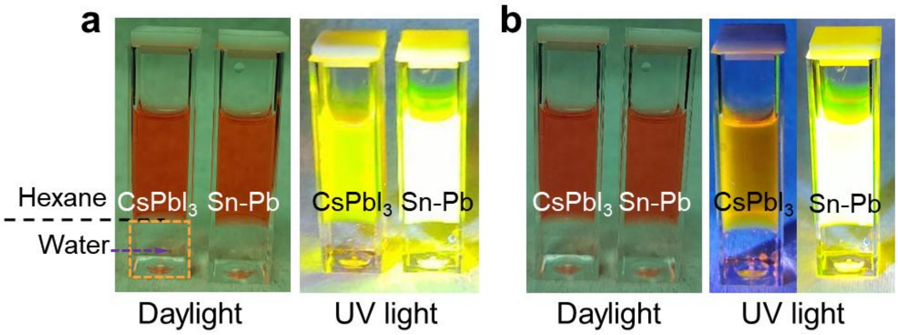

Supplementary Fig. 18 | Photographs showing the resistance of the diluted QD colloidal solutions to water treatment. (a) Fresh samples. **(b)** Aged samples with water treatment for 20 min.

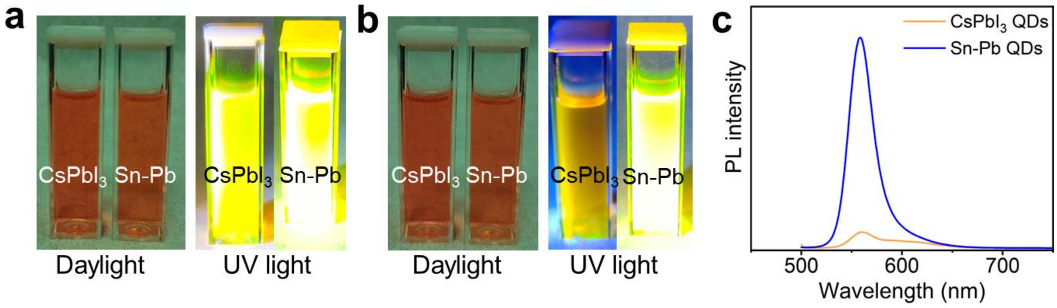

Supplementary Fig. 19 | Photographs showing the colloidal stability of the diluted $CsPbI_3$ and $CsSn_{0.09}Pb_{0.91}I_3$ QDs. (a) Fresh samples. **(b)** Aged samples for 2 h in air. **(c)** PL spectra of the aged samples. Source data are provided as a Source Data file.

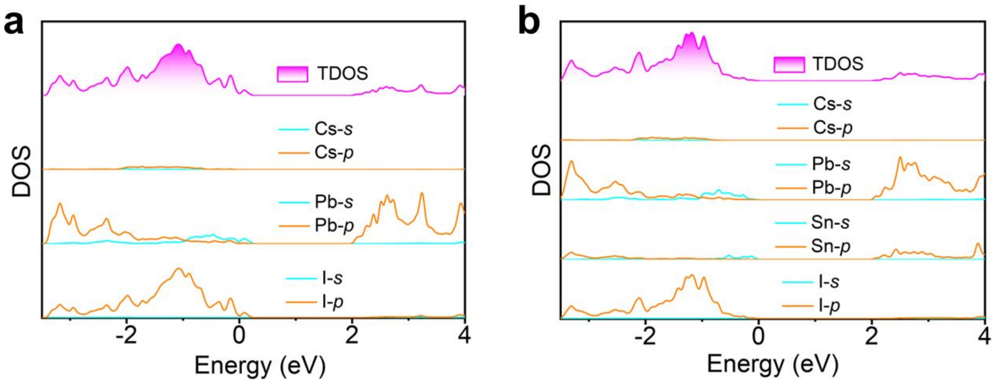

Supplementary Fig. 20 | Partial density of states (PDOS) of (a) a pristine $CsPbI_3$ slab with lead vacancy defects, and (b) a $CsPbI_3$ slab with a filled Sn^{2+}. Source data are provided as a Source Data file.

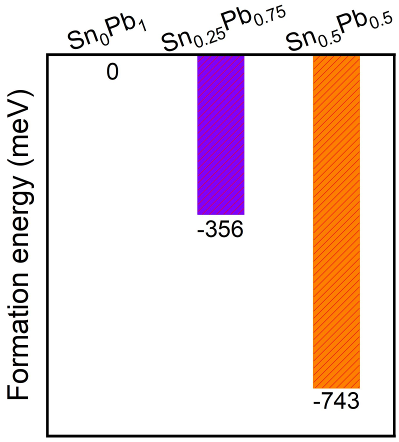

Supplementary Fig. 21 | The calculated formation energy of all-inorganic Sn-Pb iodide perovskite QDs with different contents of Sn^{2+} cations. Source data are provided as a Source Data file.

Supplementary Table 1 | PL decay parameters using bi-exponential fitting of $CsSn_xPb_{1-x}I_3$ perovskite QDs with different Pb/Sn ratio.

Sample		2:1	7:3	8:3	3:1
Fast component	τ_1 (ns)	5.52	6.15	6.55	30.46
	P_1 (%)	96.61	95.25	95.82	3.84
Slow component	τ_2 (ns)	30.60	28.88	30.91	7.14
	P_2 (%)	3.39	4.75	4.18	96.16
τ_{avg} (ns)		6.37	7.23	7.56	8.04
